# From *Şxex* to *Chorta*: The Adaptation of Maronite Foraging Customs to the Greek Ones in Kormakitis, Northern Cyprus

**DOI:** 10.3390/plants11202693

**Published:** 2022-10-12

**Authors:** Andrea Pieroni, Naji Sulaiman, Zbynek Polesny, Renata Sõukand

**Affiliations:** 1 University of Gastronomic Sciences, 12042 Bra, Italy; 2 Department of Medical Analysis, Tishk International University, Erbil 44001, Iraq; 3 Department of Crop Sciences and Agroforestry, Faculty of Tropical AgriSciences, Czech University of Life Sciences Prague, Kamýcká 129, 16500 Praha-Suchdol, Czech Republic; 4 Department of Environmental Sciences, Informatics and Statistics, Ca’ Foscari University of Venice, 30172 Venezia, Italy

**Keywords:** Cyprus, ethnobotany, Maronites, Mediterranean diet, wild greens

## Abstract

The traditional foraging of wild vegetables (WVs) has played an important role in the post-Neolithic development of rural local food systems of the Near East and the Mediterranean. This study assessed the WVs gathered by the ancient Maronite Arabic diaspora of Kurmajit/Kormakitis village in Northern Cyprus and compared them with those gathered by their Cypriot and Arab Levantine neighbors. An ethnobotanical field survey focusing on WVs was conducted via twenty-two semi-structured interviews among the few remaining Maronite elderly inhabitants (approximately 200); and the resulting data were compared with those described in a few field studies previously conducted in Cyprus, Lebanon, and coastal Syria. Wild vegetables in Kormakitis are grouped into a folk category expressed by the emic lexeme *Şxex*, which roughly corresponds to the Greek concept of *Chorta* (wild greens). The large majority of *Şxex* have Greek folk phytonyms and they overlap for the most part with the WVs previously reported to be gathered by Greek Cypriots, although a remarkable number of WVs are also shared with that of the other groups. The findings address a possible adaptation of Maronite WV foraging to the Greek one, which may be explained by the fact that the Maronite minority and the majority Greek communities lived side by side for many centuries. Additionally, after Turkish occupation in 1974, a remarkable migration/urbanization of Maronites to the main Greek centers on the southern side of the isle took place, and Kurmajit became part of Cypriot trans-border family networks.

## 1. Introduction

The Mediterranean diet (MD) was proposed for the first time in the cross-cultural epidemiological “Seven Countries Study” by the American nutritionist Ancel Benjamin Keys [1] and it concerns “food patterns typical of Crete, much of the rest of Greece, and southern Italy in the early 1960s, where adult life expectancy was among the highest in the world and rates of coronary heart disease, certain cancers, and other diet-related chronic diseases were among the lowest” [2]. This dietary system was recognized one decade ago as a UNESCO Intangible Cultural Heritage of Humanity [3] and has been described by an important spectrum of bioscientific literature, which have recently not only underlined its major health and nutrition benefits, but also its low environmental impacts, high sociocultural value, and positive local economic returns [4,5].

However, systematic detailed studies on the foraging and culinary use of wild plant ingredients in the Eastern Mediterranean and the Near East, and how these change cross-temporally and cross-culturally, are rare, despite the fact that investigating these regions is essential for better understanding the possible development and evolution of the MD. In fact, it has been proposed that the spread of the use of the wild vegetables (WVs) present in the MD may have originated in Neolithic settlements of the Fertile Crescent, where these plant resources often represented local foods and medicines [6,7].

The ethnobotany of WV foraging is, however, more generally relevant to two main environmental and social sustainability pillars: (a) community-centered sustainable management of local biodiversity resources; and (b) wild food plant cuisine heritagization; i.e., the revitalization of knowledge concerning wild plant ingredients into new educational platforms for increasing overall community botanical literacy, as well as in possible new gastronomic arenas and local-food-centered rural eco-tourism economic initiatives.

In the field of wild food ethnobotany, over the past 20 years, Cyprus has been the object of two main field studies: one conducted among the Greek population in the south [8] and one focusing on the Turkish population in the NE of the isle [9].

On the other hand, our research groups have devoted considerable effort in the past two decades to understanding how minority linguistic, ethic, and religious groups in Eastern Europe and the Near East strengthen or adapt/homogenize their identities to the majority system through wild food and medicinal plants, given the fact that these processes could be relevant to the revitalization of food or herbal heritage [7,10,11,12,13,14,15,16,17], as well as educational platforms devoted to the celebration of local bio-cultural diversity [18].

We therefore decided to conduct ethnobotanical research on WVs among the Maronites of Cyprus, who moved into the area between the 8th and 13th Centuries and still represent the totality of the population of Kurmajit/Kormakitis village [19].

The specific objectives of this research were:

(a) to record local phytonyms and traditional uses of WVs in Kormakitis; and
(b) to compare the gathered data with that collected and reported for Greek and Turkish Cyprus, Lebanon, and coastal Syria.

## 2. Materials and Methods

### 2.1. Study Village and Brief Historical Background

**Figure 1:**
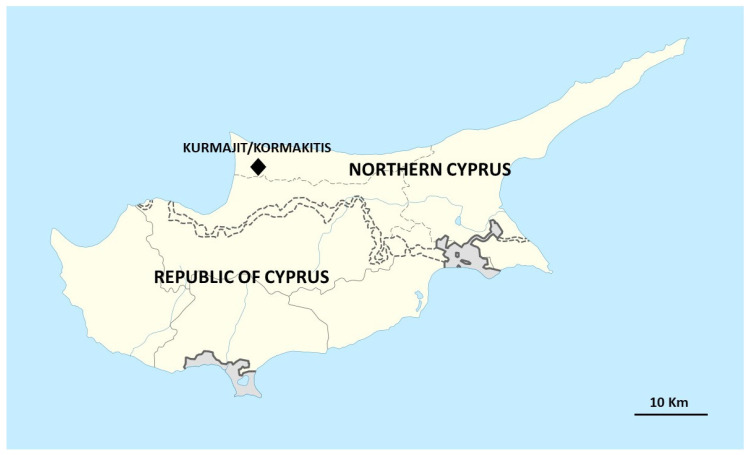
Location of Kormakitis in Cyprus.

**Figure 2:**
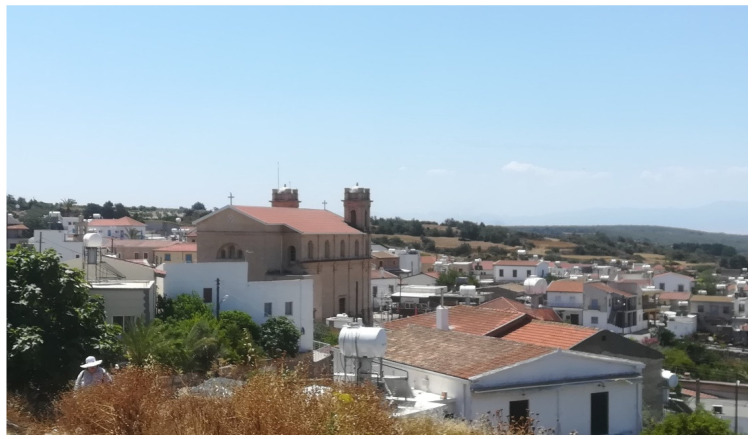
View of Kormakitis.

The field study was carried out in the small village of Kormakitis, Northern Cyprus, in May 2022 (Figure 1 and Figure 2). Most of the Maronites in Kormakitis came from a Lebanese village called Kour located in the Batroun district of Northern Lebanon [20]. The name of the studied village can be transcribed in the spoken Lebanese dialect as “Kor ma giti”, which literally means “Kour did not come”, referring to the fact that Maronites emigrated from the village of Kour, but were not able to bring the village with them; this may somehow reflect the Maronite migrants’ nostalgia for their homeland and the original Arabic identity of the village. Although Maronites represent one of the main ethno-religious groups in the Eastern Mediterranean and preserve an essential part of the region’s identity, they are still underdocumented from an ethnobotanical perspective. The uniqueness of the group is rooted in their merger of eastern Assyrian culture and rituals on one side, and the Church of Rome on the other [21]. Historically, the group originated in the Orontes Valley of present-day Syria. Between the 8th and 10th centuries, a mass migration of the group led to the transfer of the Maronite patriarchal residence to Mount Lebanon [19]. Currently, Maronites live mainly in Lebanon, where they form a quarter of the country’s population, as well as in Syria, France, USA, Mexico, and South America. Similar to other ethnic-religious groups of the region, Maronites have suffered from instability in the region over the past millennia due to various empires frequently clashing over the eastern Mediterranean (e.g., Byzantine, Persian, Roman, Umayyad, Abbasi, and Ottoman), political riots, and intricate and delicate religious affairs. Therefore, many members of the group have migrated out of the region, with Cyprus being one of the destinations [21].

**Figure 3:**
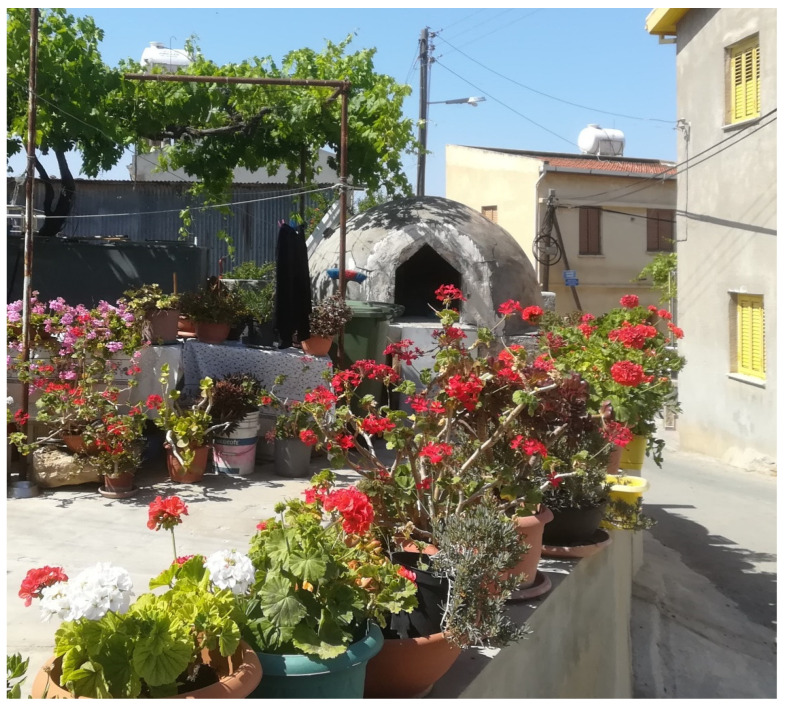
View of the village center, with private courtyard, grape pergola, and traditional external baking oven.

Kormakitis (170 m.a.s.l.) is home to approximately 300, mainly elderly, Maronite Christian inhabitants. Its surrounding rural landscape is characterized by olive orchards and the traditional local economy is based on small-scale farming. The village has a bar that serves as a gathering place for local senior citizens where they chat and play backgammon, and a family-run small restaurant famous all over the region, which serves lamb baked in the traditional Cypriot oven (*Kleftiko*) (Figure 3).

The village remained an enclave of Turkish Cyprus after division of the island in the 1970s. Those who were born there and continue living in the village receive bi-weekly food provisions by the Republic of Cyprus (Greek Cypriot) authorities via the UN troops. However, tension in the village is still high and can be sensed in the way people behave, especially in stressful situations. We witnessed a fire in a nearby village and the inhabitants were very worried because of recent experiences of help arriving late, and also possibly by signs on the streets (such as vandalized EU-support project signs), since the general feeling of the people is that they have been abandoned by Western Europeans with the exception of the Catholic Pope. The inhabitants, especially the elderly, speak a specific dialect of Arabic (Cypriot Maronite Arabic) as their mother tongue while being fluent in Greek and Turkish as well. An elderly woman recalled that Arabic was their cultural savior after the Turkish occupation, and Kormakits is the only active and entirely Christian village in present-day Northern Cyprus.

### 2.2. Ethnobotanical Field Study

The ethnobotanical field study was carried out in Kormakitis in May 2022. The main purpose of the survey was to record local knowledge of wild vegetables currently gathered and consumed by locals. Twenty-two elderly residents (range: 64 to 84 years old), especially rural farmers and elderly women who were considered potential WV local knowledge holders in the area, were recruited through snowball techniques to participate in semi-structured interviews. Prior to each interview, verbal consent was obtained from each of the participants and the Code of Ethics adopted by the International Society of Ethnobiology was followed [22]. Full anonymity was observed during the interviews. For each of the WVs free listed during the study, the local name and local food uses were documented. We deliberately excluded from the survey other wild food plants, such as wild fruits. The quoted wild food taxa were collected from the study area, when available, and identified by the authors using standard reference works and checklists [23,24].

Voucher specimens (bearing the accession code UVVETBOT) were deposited at the Herbarium of the Biocultural Diversity Lab of the Department of Environmental Sciences, Informatics and Statistics, Ca’ Foscari University of Venice, Italy. The identification of wild plants that were not available during the field study was conducted on the basis of folk names and detailed plant descriptions; in this case, pictures of the presumed plants were shown to the study participants after a preliminary evaluation of the quoted folk name and description. Nomenclature always followed The World Flora Online database [25], while plant family assignments were consistent with the Angiosperm Phylogeny website [26]. Recorded local Arabic plant names were reported in Romanized transliterations following standard reference works [27].

### 2.3. Data Analysis

A historical comparison was conducted in May–August 2022, analyzing the data gathered in the current study together with those reported by three food ethnobotanical studies conducted in the past two decades in Cyprus among Greeks [8] and Turks [9], Lebanon [28], and coastal Syria [29]. In addition, we used both our observations and unpublished data from the field work conducted in coastal Syria for qualitative interpretation of our present data from Kormakitis.

## 3. Results

We recorded the use of twenty-nine folk plant taxa used as leafy vegetables, corresponding to thirty-six botanical species (Table 1). The most represented family was Asteraceae (twelve species) and the use of eight genera (*Asparagus*, *Capparis*, *Crithmum*, *Cynara*, *Foeniculum*, *Glebionis*, *Portulaca*, and *Sinapis*) was named by more than 40% of interviewees.

**Figure 4:**
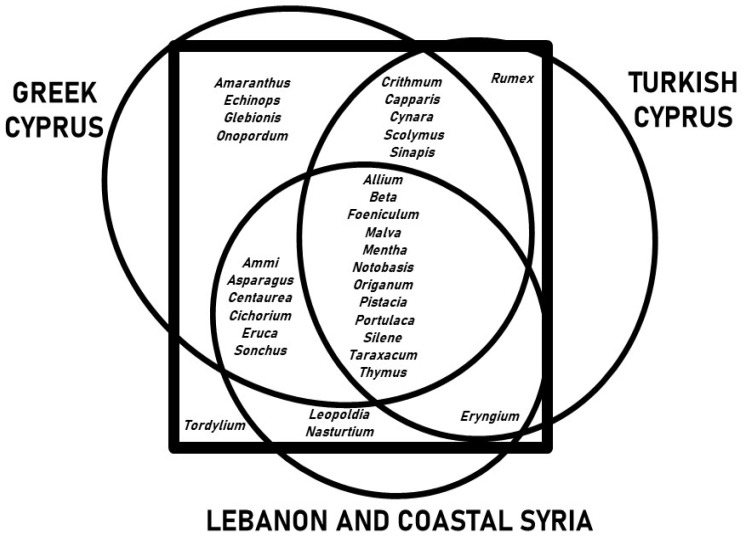
Venn diagram showing the overlap between the wild vegetables gathered and consumed in Kormakitis, and those recorded among Greek and Turkish Cypriots, and among Arabs in Lebanon and coastal Syria.

Figure 4 shows the overlap between the WVs gathered and consumed in Kurmajit, and those of their Greek and Turkish neighbors and communities in Lebanon and Syria. The highest number of overlaps (twenty-eight out of thirty-two genera) are with the records from Greek Cyprus, four of them exclusively (*Amaranthus*, *Echinops*, *Glebionis*, *Onopordum*). Of the eighteen genera shared with Turkish Cyprus, only one taxon (*Rumex*) was shared exclusively, while twenty-one taxa were also used in Lebanon and coastal Syria. The only taxon found exclusively in the Maronite community was *Tordylium*, which was used as a seasoning.

**Figure 5:**
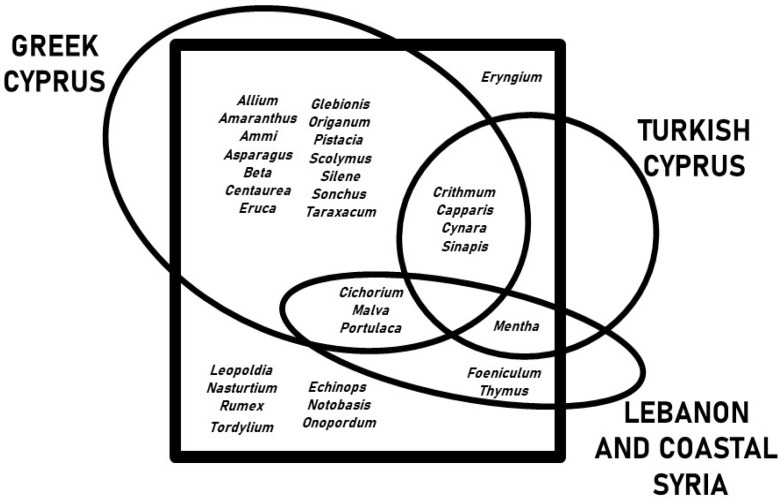
Venn diagram showing the overlaps between the folk names of wild vegetables in Kormakitis and those recorded among Greek and Turkish Cypriots, and among Arabs in Lebanon and coastal Syria.

Figure 5 shows the overlap of Cypriot Maronite folk plant names and those referring to WVs documented among Cypriot Greeks and Turks, as well as among Arabs in Lebanon and coastal Syria. The overlap with Greek Cypriot phytonyms is remarkable and includes approximately two thirds of the recorded folk plant names.

## 4. Discussion

Wild vegetables in Kormakitis are grouped into a folk category expressed by the lexeme *Şxex*, which roughly corresponds to the Greek concept of *Chorta*; i.e., wild greens. Locals regard the bitter, weedy Asteraceae, i.e., *Cichorium*, *Taraxacum*, and *Sonchus* spp., as prototypical for *Şxex*. The local name *Şxex* could possess some Arabic roots as the word *Skhakh* (سخاخ) means “the free soft land”, which somehow refers to the wild. The Maronites in coastal Syria refer to wild greens as *Mhabbleh*, which literally means “the steamed food” [29]; however, another local name for wild leafy vegetables in coastal Syria is *Sleeq*, which overlaps in some phonemes with *Şxex*. Nonetheless, the large majority of the WVs traditionally foraged by Maronites have Greek folk names and they overlap for the most part with the WVs previously reported to be gathered by Greek Cypriots. Only minor overlaps with Lebanese and Turkish Cypriot WV ethnobotanies were found; for instance, overlap with Arabic names was found in the species *Foeniculum vulgare*, *Thymus capitatus*, and *Mentha spicata*. On the other hand, Table 1 shows similarities in the local names of a few species (*Portulaca oleracea*, *Malva sylvestris*, *Cichorium intybus*) between our study participants in Kormakitis and the Greek minority in coastal Syria. The findings from Kormakitis, however, show a remarkable adaptation of possible original Maronite WV foraging customs to Greek Cypriot ones. This finding can be explained by the fact that the Maronites of Kormakitis lived together with their Greek neighbors for several centuries, although in the past intermarriages between the two communities were nearly impossible (Maronites are Catholic Christians, while Greeks are Orthodox Christians). This means that before the Turkish invasion of Northern Cyprus in 1974, original Maronite customs and thus, plant foraging, may have been “hellenicized”.

In contrast, minimal influences from Turkish Cypriot WV ethnobotany may have played a role only recently, after Kormakitis and Northern Cyprus were occupied approximately five decades ago. While the use of *Rumex* was borrowed from Turkish Cyprus, they still maintained a distinct name for it, which may indicate they had some other need for differentiating this plant.

However, the custom of foraging seems to have disappeared in Kormakitis among the middle-aged generation; this, apart from global trends, may be due to the fact that after Turkish occupation, a remarkable migration/urbanization of Maronites from Kormakitis and other Northern Cypriot villages to the main Greek centers on the southern side of the isle took place, with extended families becoming transnational and commuting more or less regularly. Even now, individuals are moving back to the village; thus, further enhancing “urbanization” and the dilution of the village’s foraging heritage.

Finally, the fact that the village has been, and continues to be, supported by the Republic of Cyprus (Greek Cypriot) authorities, who, via UN troops, deliver food (along with water, fuel, and medical supplies) across the border every two weeks, may have diluted the local need for foraged foods during the past few decades.

## 5. Conclusions

Plant biodiversity and the traditional/local ecological knowledge attached to it are essential for the holistic sustainability of “socio-ecological systems”. This study suggests that the recorded Maronite wild vegetables could provide crucial baseline data for revitalizing local wild plant knowledge and practices in educational arenas, as well as their sustainable foraging, ideally aimed at promoting local food heritage and the community-centered management of plant resources.

## Figures and Tables

**Table 1:** Recorded Cypriot Maronite wild vegetables in Kormakitis, including their folk names, local culinary uses, frequency of quotation, and comparison to reports from Lebanon, coastal Syria, and Greek and Turkish Cyprus.

Botanical Taxon or Taxa, Botanical Family; Voucher Specimen Code (UVVETBOT)	Local Name(s)	Used Parts	Local Food Uses	Frequency of Quotation	Folk Names and Frequency of Quotation in Lebanon	Folk Names and Frequency of Quotation in Coastal Syria	Folk Names and Frequency of Quotation in Greek Cyprus	Folk Names in Turkish Cyprus
*Allium ampeloprasum* L., Amaryllidaceae; Cr01	**Agrioskordo, Skordo**	Whole plant	Salads, cooked, seasoning	+	Kurrat +++	Kerrat +++	Agriopraso, **Agrioskordo** +	Yabani pırasa
*Amarantus album* L., Amaranthaceae; Cr23	**Ghlindo**	Leaves	Cooked, especially with beans	++			**Glindos**, Vlito +	
*Ammi majus* L., Apiaceae	**Agriosellino**	Aerial parts	Salads	+		Khelleh +	**Arkoseleno** +	
*Asparagus acutifolius* L., Asparagaceae; CY09	**Agrelia, Agrenli**	Young shoots	Boiled or cooked with eggs	+++	Halyoun ++	Halyoun +++	**Agrelia** +	
*Beta vulgaris* L., Amaranthaceae; CY10	**Agriolahano, Lahanutkia**	Leaves	Boiled	+		Selq barri +	**Agriolachano**, Agrioteflo +	Yabani ıspinak, Pazı
*Capparis spinosa* L., Capparaceae; CY02	**Kappari**	Flower buds, unripe fruits, and young aerial parts	Pickled or in mixed salads	+++			**Kappari** +++	**Gabbar**
*Centaurea calcitrapa* L., Asteraceae; Cr78	**Trisaġia**	Young rosettes	Cooked, especially with beans	++	Dardar, Dardrieh ++	Dardar, Qellaibeh ++	Agratsia, Atrachia, Atrachouna, **Trisatsia** +++	
*Cichorium intybus* L., Asteraceae; Cr97	**Radiċa**, Vallişxex	Rosettes	In mixed salads or boiled	+	Aalet, Barrieh, Hindbeh +++	Hendbeh +++	**Agrioradiki**a ++	
*Crithmum maritimum* L., Apiaceae, CY74	**Kirdama, Kirtam**	Young aerial parts	Boiled, then pickled	+++			**Kirtamo** +	Deniz otu, **Girtama**
*Cynara cornigea* Lindl. and *C. cardunculus* L., Asteraceae; CY54 and CY58	**Agriaġinara, Anġinares, Hostos,** **Karkarua**	Flower receptacles and stems	Raw (consumed with lemon and salt), pickled, cooked	+++			**Agrioagkinara, Arzotsinara, Chosti, Kafkarua** **+**	**Hostes**, Gafgarit
*Echinops spinossimum* Turra, Asteraceae; CY01	Şerabit, Şrabir, Şrabit	Young stems	Raw as snacks or in salads	+			Saratzinos +	
*Eruca vesicaria* (L.) Cav., Brassicaceae; CY38	**Roxa**	Leaves	Salads	+	Jarjeer ++		**Roca** +	
*Eryngium campestre* L., Asteraceae; CY25	**Bangallo, Pangaros**	Tender upper roots and shoots	Pickled	++	Aarakbeen, Jibaaneh, Quraset el aaneh, Qursaneeh +++			Kazayaği, **Mangallo**
*Foeniculum vulgare* Mill., Apiaceae; Cr09, Cr19	**Şummar**	Aerial parts	Raw, cooked, seasoning (especially snails)	**+++**	**Shumar, Shumra** **+++**	Shamra ++	Marathos ++	Dere otu, Maraho, Rezene
*Glebionis segetum* Fourr., Asteraceae; CY07	Lazaros, **Similia**	Young shoots	Raw, as a snack and in salads	+++			**Similia** +	
*Leopoldia comosa* (L.) Parl., Asparagaceae; Cr75	Arkoskorto	Bulbs	Cooked in various ways, pickled	++	Agriohyacinthos +			
*Malva sylvestris* L., Malvaceae; CY08	**Moloxa**	Leaves	Boiled	++	Khubbaizeh +++	Khebbaizeh **+++**	**Molocha, Molochoua** ++	Gömeç
*Mentha spicata* L., Lamiaceae; CY45	**Nana**	Young leaves	Seasoning	++	**Naana barri** +++		Dyosmos +	**Dere nanesi, Yabani nana**
*Nasturtium officinale* W. T. Aiton, Brassicaceae; Cr34	Gardamo	Aerial parts	Salads	+		Jarjeer +++		
*Notobasis syriaca* (L.), Cass., Asteraceae; CY29	Şerabit, Şrabir, Şrabit	Young stems	Raw as snacks or in salads	+		Kherfesh, Qailouh, Shok aljamal ++	Gauroukavlos, Nerokavlos, Patsalokavlos +	Sūtleğen, Tuzlu gavulya
*Onopordum cyprium* Eig, Asteraceae; CY19	Şerabit, Şrabir, Şrabit	Young stems	Raw as snacks or in salads	+			AsGaedouragkatho +	
*Origanum syriacum* (Boiss.) Kuntze and *O. majorana* L., Lamiaceae	**Rigani**	Flowering aerial parts	Seasoning	**+**	Zaatar +++	Zauba’ +++	**Rigani,** Sapsissia **+**	Dağ kekiği
*Pistacia terebinthus* L. Anacardiaceae; CY04	**Tremi**θia	Fruits	Seasoning sausages	+	Shaashoub ++	Betem ++	**Tremithia** **++**	Çitlembic, Menengiç
*Portulaca oleracea* L. aggr., Portulacaceae; CY11	**Glistirìa, Glistriδa**, Nistri**δ**a	Aerial parts	Salads	+++	Baqleh, Farfahin +++	Beqail barriah **++**	**Glystirida** +	Semiz otu
*Rumex crispus* L., Polygonaceae; CY8	**Laxana**, Xamedui, Zamedui	Leaves	Cooked, dolmades	+				Labada, Yabani ıspanak
*Scolymus hispanicus* L., Asteraceae; CY25	**Zalatuna**	Young shoots, tender peduncles, and rachis of leaves (sometimes with parts of the stem); underground part of stems and external coat of the roots	Cooked	++			Alatouna, Aspragkatho, Atrachounes, Christagkatho, Galaktites, **Galatouna,** Plotarka +++	Kara diken, Kara ot, Otluk, Sahura, Saracino
*Silene vulgaris* (Moench) Garcke, Caryophyllaceae; CY75	**Stru**θ**kia**	Young shoots	Cooked	+	Dwaylineh +		**Stroufouthkia**, **Strouthi**, Tsakridia +	Gıcır, Kuş otu, Serçe otu, Yumurta tu
*Sinapis alba* L. and *S. arvensis* L., Brassicaceae; CY25 and CY26	**Lapsana**	Young aerial parts	Boiled	+++			**Lapsana** +	**Lapsana**, Hardal
*Sonchus oleraceus* L., Asteraceae; CY12	Radi**ċ**a, **Şxex**	Young aerial parts	In mixed salads or boiled	+		Khesaiseh, Asat alraa’i, Elk alghazal ++	**Sonchos,** Tsiofos, **Tsionchos** ++	
*Taraxacum cyprium* H.Lindb. and *T. hellenicum* Dahlst., Asteraceae; CY23 and Cr07	**Pikraradiċa, Radiċa morren, Şxex**	Young aerial parts	In mixed salads or boiled	+	Chissaisseh ++		**Agriodrakia, Agrioraditzia** +	Karahindiba
*Thymus capitatus* (L.) Hoffmanns. & Link, Lamiaceae; Cr13	**Zaatar**	Flowering tops and leaves	Seasoning	+	**Zaatar** +		Throuembi, Thymari +++	
*Tordylium apulum* L., Apiaceae; Cr02	Miriaθeo	Young aerial parts	Seasoning	++				

Frequency of quotation: +++: quoted by 40–100% of the study participants; ++: quoted by 10–39% of the study participants; +: quoted by less than 10% of the study participants. Folk linguistic cognates are reported in bold.

## Data Availability

Not applicable.
